# Supplementary figures and images for: RSC and GRFs confer promoter directionality by restricting divergent noncoding transcription
Source: Life Sci Alliance. 2022 Sep 16;5(12):e202201394. doi: 10.26508/lsa.202201394 (PMC9481977; doi:10.26508/lsa.202201394)

Figure S1A

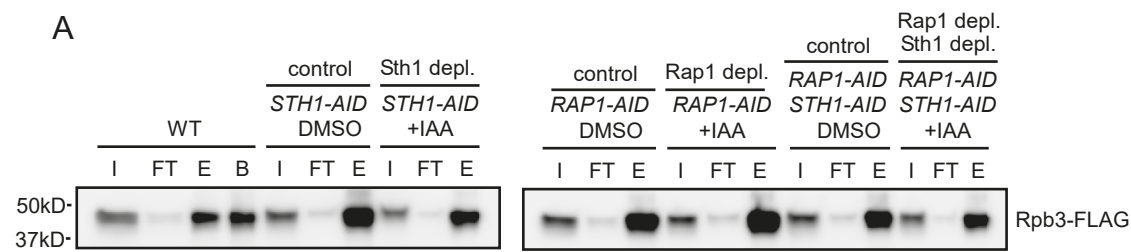

Rpb3-FLAG

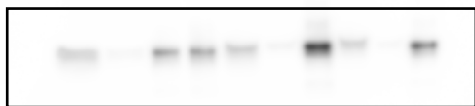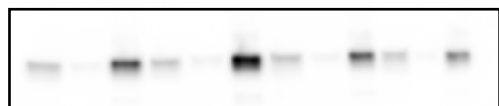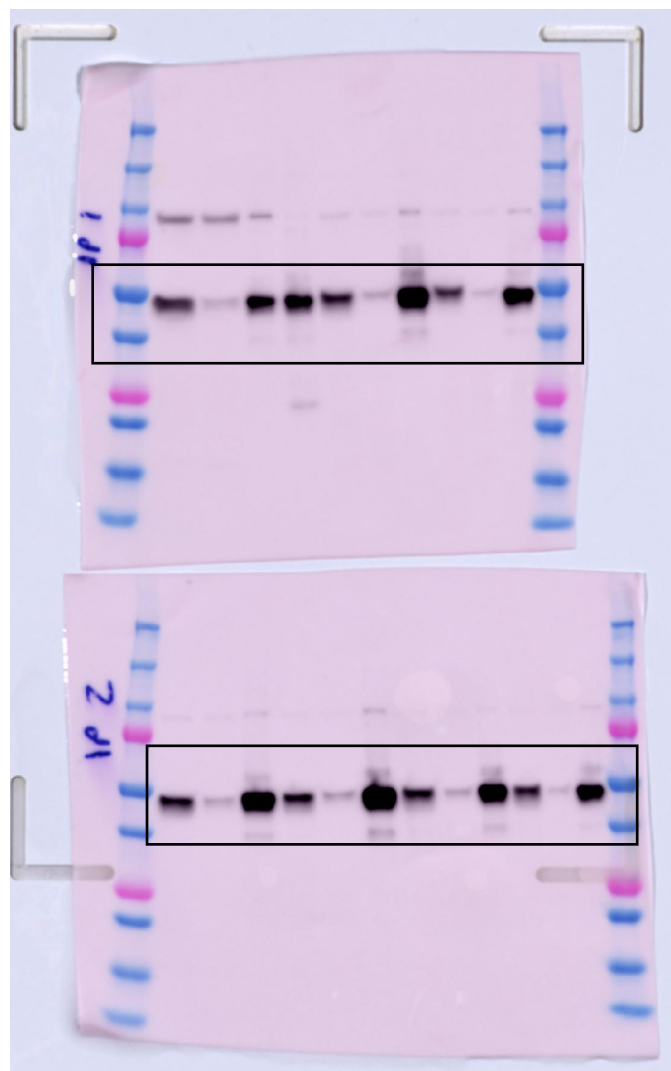

Supplement: Supplementary file 1 [file LSA-2022-01394_SdataFS1.pdf]

Figure 1A

A

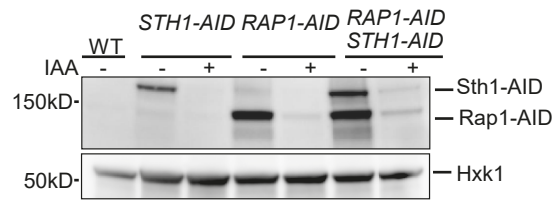

Sth1-AID  
Rap1-AID

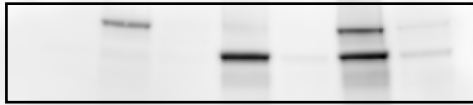

250kD  
150kD  
100kD  
75kD  
50kD  
37kD  
25kD  
20kD

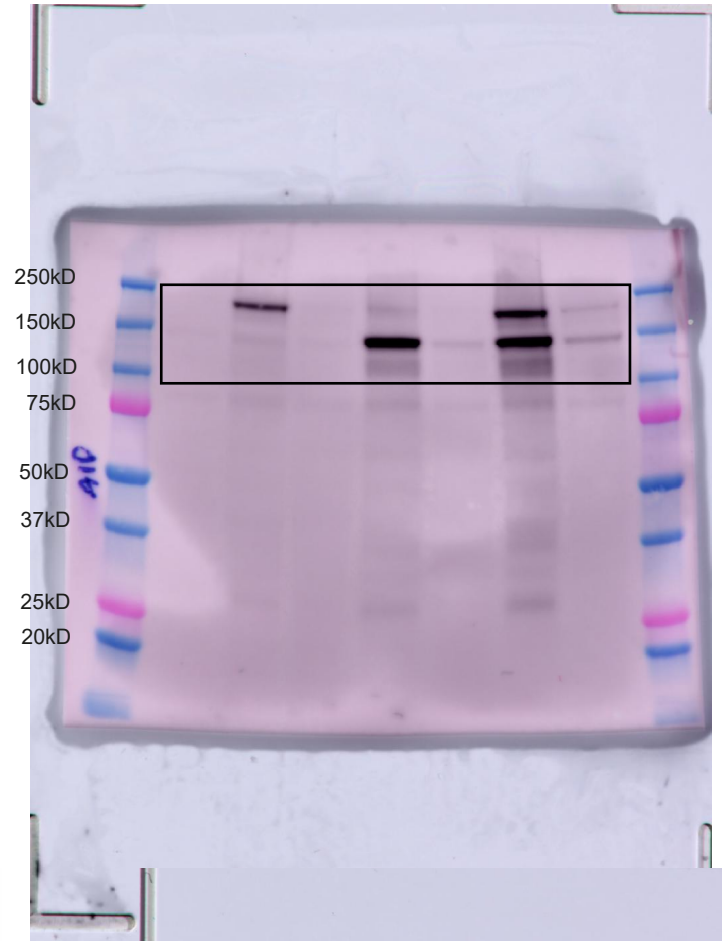

Hxk1

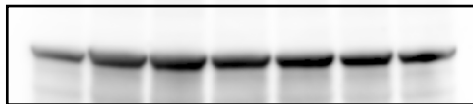

250kD  
150kD  
100kD  
75kD  
50kD  
37kD  
25kD  
20kD

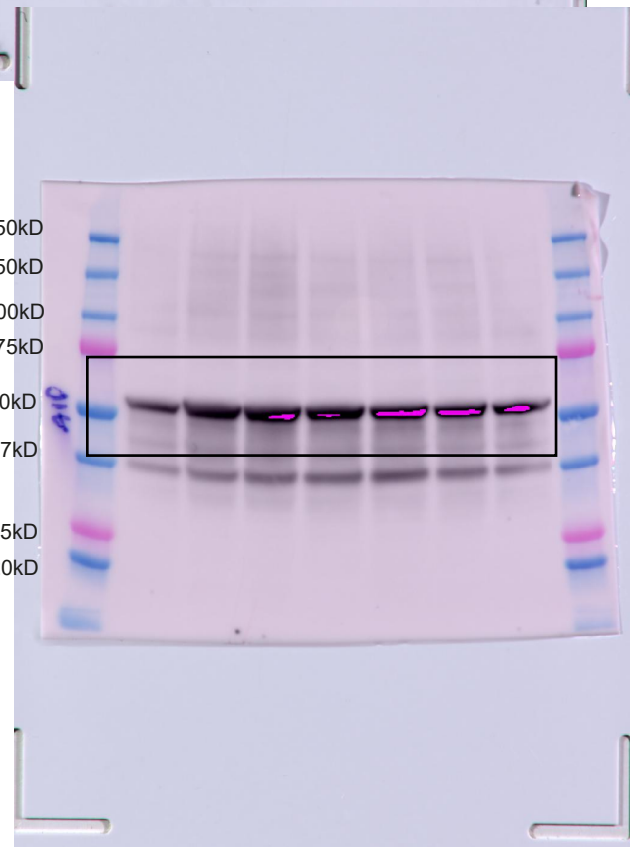

Supplement: Supplementary file 2 [file LSA-2022-01394_SdataF1.pdf]

Figure 6G

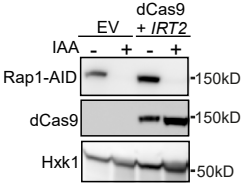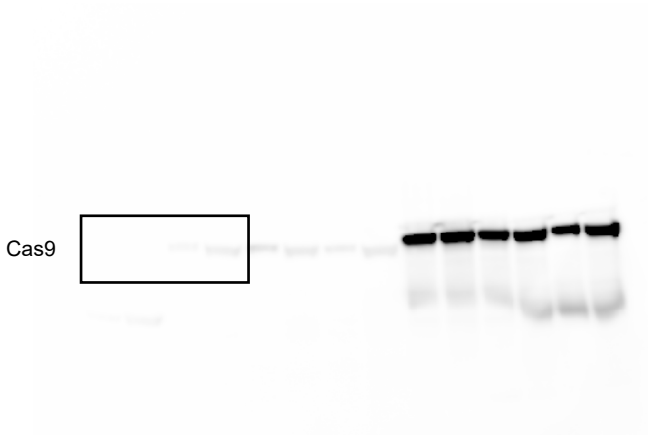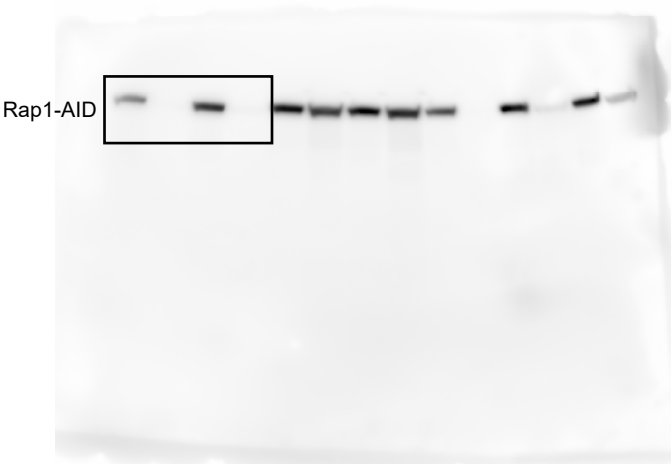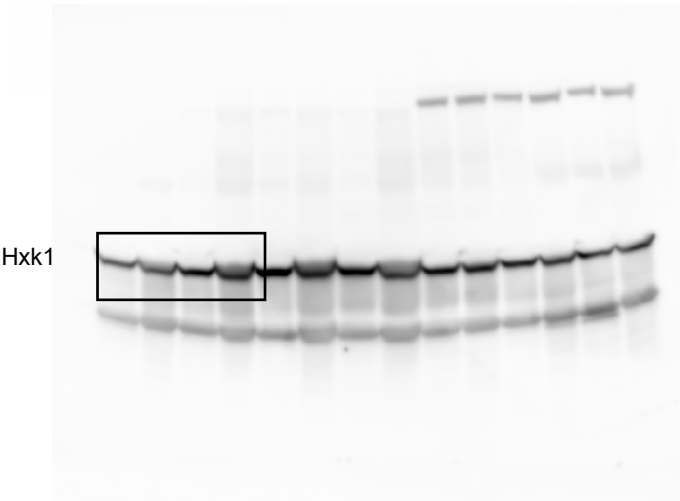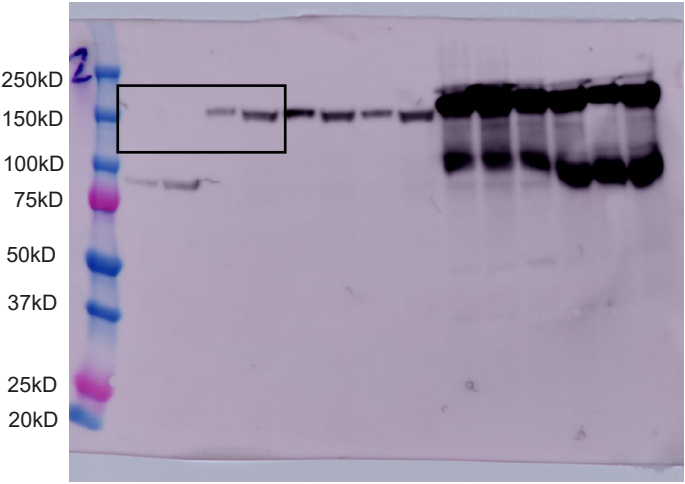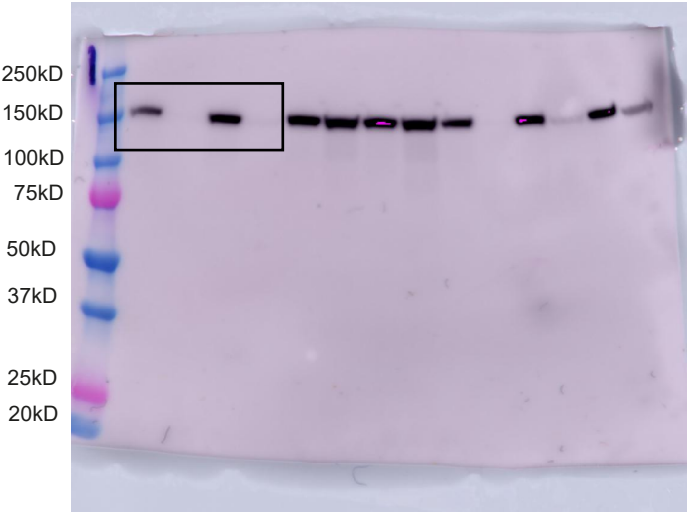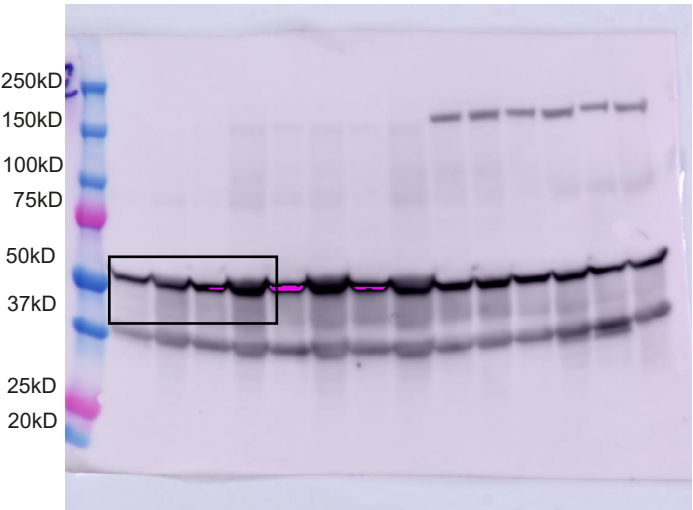

Figure 6H

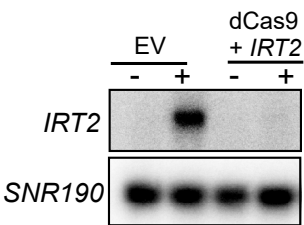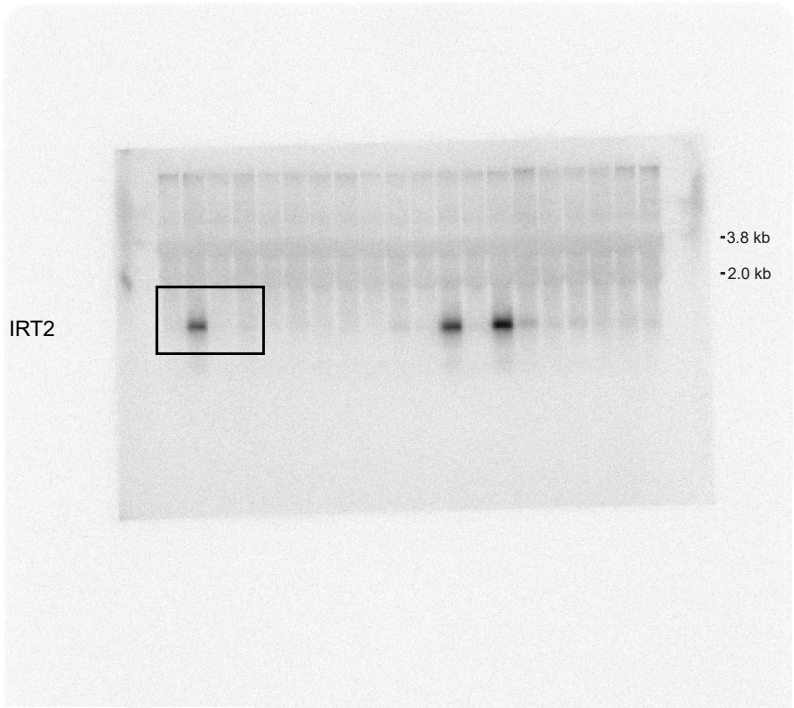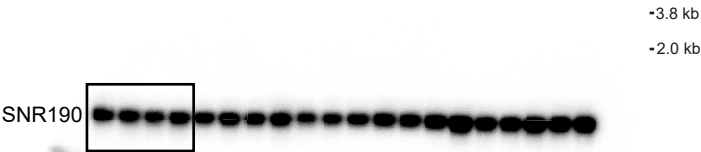

Supplement: Supplementary file 4 [file LSA-2022-01394_SdataF6.pdf]

Figure S6B

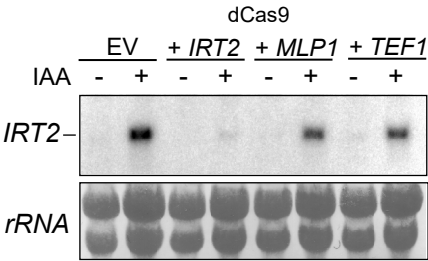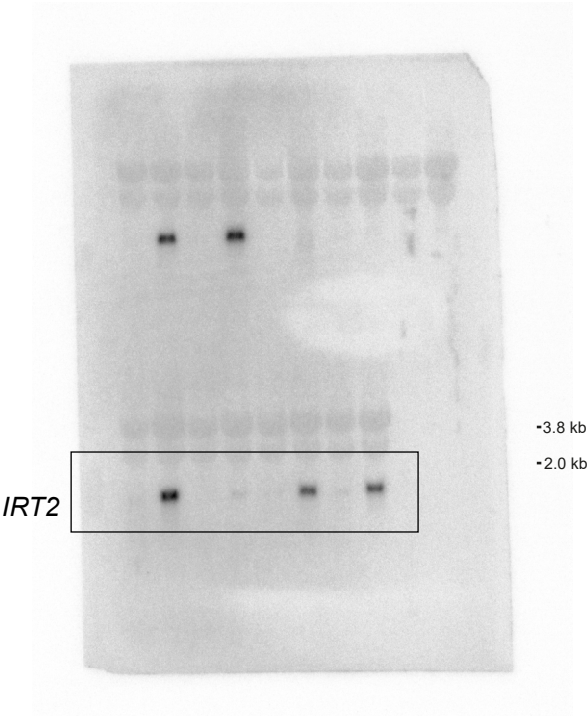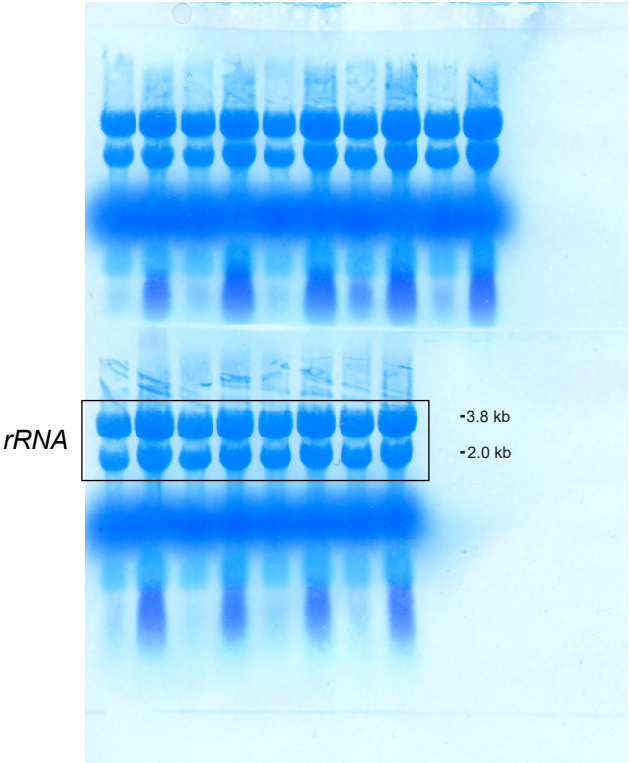

Supplement: Supplementary file 5 [file LSA-2022-01394_SdataFS6.pdf]
