# Supplementary material for: RSC and GRFs confer promoter directionality by restricting divergent noncoding transcription
Source: Life Sci Alliance. 2022 Sep 16;5(12):e202201394. doi: 10.26508/lsa.202201394 (PMC9481977; doi:10.26508/lsa.202201394)
Supplement: Supplementary file 6 [file LSA-2022-01394_TableS2.pdf]

Table S2. Genotypes of strains used in this study

Strain Number	Genotype
FW7228	*MATa his3Δ1 leu2Δ0 met15Δ0 ura3Δ0 rpb3::RPB3-3xFLAG::NATMX*
FW7220	*MATa his3Δ1 leu2Δ0 met15Δ0 ura3Δ0 rpb3::RPB3-3xFLAG::NATMX leu2::pGPD1-osTIR1::LEU2 sth1::STH1-V5-AID::KANMX6*
FW7232	*MATa his3Δ1 leu2Δ0 met15Δ0 ura3Δ0 rpb3::RPB3-3xFLAG::NATMX leu2::pGPD1-osTIR1::LEU2 rap1::RAP1-V5-AID::KANMX6 sth1::STH1-V5-AID::KANMX6*
FW7238	*MATa his3Δ1 leu2Δ0 met15Δ0 ura3Δ0 rpb3::RPB3-3xFLAG::NATMX leu2::pGPD1-osTIR1::LEU2 rap1::RAP1-V5-AID::KANMX6*
FW6404	*MATa his3Δ1 leu2Δ0 met15Δ0 ura3Δ0 ppt1::p615-YFP-Cbf1_bs-pPPT1-mCherry::NATMX6*
FW6306	*MATa his3Δ1 leu2Δ0 met15Δ0 ura3Δ0 cbf1::KANMX ppt1::p615-YFP-Cbf1_bs-pPPT1-mCherry::NATMX6*
FW6401	*MATa his3Δ1 leu2Δ0 met15Δ0 ura3Δ0 ppt1::p612-YFP-Gcn4_bs-pPPT1-mCherry::NATMX6*
FW6300	*MATa his3Δ1 leu2Δ0 met15Δ0 ura3Δ0 gcn4::KANMX ppt1::p612-YFP-Gcn4_bs-pPPT1-mCherry::NATMX6*
FW6402	*MATa his3Δ1 leu2Δ0 met15Δ0 ura3Δ0 ppt1::p613-YFP-Cat8_bs-pPPT1-mCherry::NATMX6*
FW6302	*MATa his3Δ1 leu2Δ0 met15Δ0 ura3Δ0 cat8::KANMX ppt1::p613-YFP-Cat8_bs-pPPT1-mCherry::NATMX6*
FW6403	*MATa his3Δ1 leu2Δ0 met15Δ0 ura3Δ0 ppt1::p614-YFP-Gal4_bs-pPPT1-mCherry::NATMX6*
FW6424	*MATa his3Δ1 leu2Δ0 met15Δ0 ura3Δ0 gal4::KANMX ppt1::p614-YFP-Gal4_bs-pPPT1-mCherry::NATMX6*
FW6405	*MATa his3Δ1 leu2Δ0 met15Δ0 ura3Δ0 ppt1::p616-YFP-Gcr1_bs-pPPT1-mCherry::NATMX6*
FW6315	*MATa his3Δ1 leu2Δ0 met15Δ0 ura3Δ0 gcr1::KANMX ppt1::p616-YFP-Gcr1_bs-pPPT1-mCherry::NATMX6*
FW6415	*MATa his3Δ1 leu2Δ0 met15Δ0 ura3Δ0 abf1::ABF1-V5-IAA7::KANMX6 leu2::pGPD1-osTIR1::LEU2 ppt1::p619-YFP-Abf1_bs-pPPT1-mCherry::NATMX6*

FW6411	*MATa his3Δ1 leu2Δ0 met15Δ0 ura3Δ0*<br>*reb1::REB1-V5-IAA7::KANMX6 leu2::pGPD1-osTIR1::LEU2*<br>*ppt1::p620-YFP-Reb1_bs-pPPT1-mCherry::NATMX6*
FW6407	*MATa his3Δ1 leu2Δ0 met15Δ0 ura3Δ0*<br>*ppt1::p592-YFP-pPPT1-mCherry::NATMX6*
FW629	*MATa his3Δ1 leu2Δ0 met15Δ0 ura3Δ0*
FW8477	*MATa his3Δ1 leu2Δ0 met15Δ0 ura3Δ0*<br>*rap1::RAP1-V5-IAA7::KANMX6 his3::pGPD1-osTIR1::HIS3*<br>*ura3::p375::URA3*
FW8523	*MATa his3Δ1 leu2Δ0 met15Δ0 ura3Δ0*<br>*rap1::RAP1-V5-IAA7::KANMX6 his3::pGPD1-osTIR1::HIS3*<br>*ura3::p704::pTDH3-dCas9-3xFLAG-ADH1term::URA3*<br>*p692::pRS305-pSNR52-sgIRT2*
FW8531	*MATa his3Δ1 leu2Δ0 met15Δ0 ura3Δ0*<br>*rap1::RAP1-V5-IAA7::KANMX6 his3::pGPD1-osTIR1::HIS3*<br>*ura3::p703::pTDH3-dCas9-Mxi1-3xFLAG-ADH1term::URA3*<br>*p692::pRS305-pSNR52-sgIRT2*
FW8527	*MATa his3Δ1 leu2Δ0 met15Δ0 ura3Δ0*<br>*rap1::RAP1-V5-IAA7::KANMX6 his3::pGPD1-osTIR1::HIS3*<br>*ura3::p703::pTDH3-dCas9-3xFLAG-ADH1term::URA3*<br>*p705::pRS305-pSNR52-sgTEFI*
FW8535	*MATa his3Δ1 leu2Δ0 met15Δ0 ura3Δ0*<br>*rap1::RAP1-V5-IAA7::KANMX6 his3::pGPD1-osTIR1::HIS3*<br>*ura3::p703::pTDH3-dCas9-Mxi1-3xFLAG-ADH1term::URA3*<br>*p352::pRS305-pSNR52-sgTEF1*
FW8529	*MATa his3Δ1 leu2Δ0 met15Δ0 ura3Δ0*<br>*rap1::RAP1-V5-IAA7::KANMX6 his3::pGPD1-osTIR1::HIS3*<br>*ura3::p703::pTDH3-dCas9-Mxi1-3xFLAG-ADH1term::URA3*<br>*p706::pRS305-pSNR52-sgMLP1*
