# Supplementary material for: RSC and GRFs confer promoter directionality by restricting divergent noncoding transcription
Source: Life Sci Alliance. 2022 Sep 16;5(12):e202201394. doi: 10.26508/lsa.202201394 (PMC9481977; doi:10.26508/lsa.202201394)
Supplement: Supplementary file 7 [file LSA-2022-01394_TableS3.pdf]

Table S3. Plasmids used in this study.

plasmid number	plasmid name
592	*YFP-pPPT1-mCherry::NATMX6*
615	*YFP-Cbf1_bs-pPPT1-mCherry::NATMX6*
612	*YFP-Gcn4_bs-pPPT1-mCherry::NATMX6*
613	*YFP-Cat8_bs-pPPT1-mCherry::NATMX6*
614	*YFP-Gal4_bs-pPPT1-mCherry::NATMX6*
616	*YFP-Gcr1_bs-pPPT1-mCherry::NATMX6*
619	*YFP-Abf1_bs-pPPT1-mCherry::NATMX6*
620	*YFP-Reb1_bs-pPPT1-mCherry::NATMX6*
706	*pRS305-pSNR52-sgMLP1*
247	*pL243 (plasmid expression TIR1 ligase)*
227	*pWG444 (plasmid for making NAT deletion)*
627	*3xFLAG-NATMX*
704	*pTDH3-dCas9-3xFLAG-ADH1term*
703	*pTDH3-dCas9-Mxi1-3xFLAG-ADH1term*
692	*pRS305-pSNR52-sgIRT2*
352	*pRS305-pSNR52-sgTEF1*
252	*pFA6A-V5-IAA7::KANMX6*
