# Supplementary material for: RSC and GRFs confer promoter directionality by restricting divergent noncoding transcription
Source: Life Sci Alliance. 2022 Sep 16;5(12):e202201394. doi: 10.26508/lsa.202201394 (PMC9481977; doi:10.26508/lsa.202201394)
Supplement: Supplementary file 8 [file LSA-2022-01394_TableS4.pdf]

Table S4. Oligo nucleotide sequences used in this study

IME1_NB_probe_R	ATGCAACGCCTACTTGTTTT	*IRT2* northern blot probe
IME1_NB_probe_F	GATGGAGGGTTGGCATAAAA	*IRT2* northern blot probe
SNR190_NB_probe_F	GGCCCTGATGATAATG	*SNR190* northern blot probe
SNR190_NB_probe_R	GGCTCAGATCTGCATG	*SNR190* northern blot probe
IRT2_RT_F	GACATCCGCATTCTTGCAGC	*IRT2* for RT-qPCR
IRT2_RT_R	CATGCTGTTCTTTCCGCCAC	*IRT2* for RT-qPCR
ACT1_RT_F	GTACCACCATGTTCCCAGGTATT	*ACT1* RT-qPCR
ACT1_RT_R	AGATGGACCACTTTCGTCGT	*ACT1* RT-qPCR
IRT2_gRNA_F	**ATGATATGTAG**TTTTAGAGCTAGAAATAGCAAGTTAAA	*IRT2* gRNA primer for cloning, highlighted is the gRNA sequence used
IRT2_gRNA_R	**TTAGATTATT**GATCATTTATCTTTCACTGCGGA	*IRT2* gRNA primer for cloning, highlighted is the gRNA sequence used
MLP1_gRNA_F	**GAGGCACGGAG**TTTTAGAGCTAGAAATAGCAAGTTAAA	*MLP1* gRNA primer for cloning, highlighted is the gRNA sequence used
MLP1_gRNA_R	**TAATAGATTT**GATCATTTATCTTTCACTGCGGA	*MLP1* gRNA primer for cloning, highlighted is the gRNA sequence used
Reb1bs_top	TCGGGTAACTTATCGGGTAACAT	Oligo used for cloning Reb1 binding site
Reb1bs_bottom	ATGTTACCCGATAAGTTACCCGA	Oligo used for cloning Reb1 binding site
Abf1bs_top	TTATCACTTCCCACGATTATCACTTCCCACGATT	Oligo used for cloning Abf1 binding site
Abf1bs_bottom	AATCGTGGGAAGTGATAATCGTGGGAAGTGATAA	Oligo used for cloning Abf1 binding site
Gal4bs_top	ACGGATTAGAAGCCGCCGAGCGGGCGACAGCCCTCCGACGGAAGACTCTCCTCCGT	Oligo used for cloning Gal4 binding site
Gal4bs_bottom	ACGGAGGAGAGTCTTCCGTCGGAGGGCTGTCGCCCGCTCGGCGGCTTCTAATCCGT	Oligo used for cloning Gal4 binding site

Gcn4bs_top	TATGACTCATTCTATGACTCATTC	Oligo used for cloning Gcn4 binding site
Gcn4bs_bottom	GAATGAGTCATAGAATGAGTCATA	Oligo used for cloning Gcn4 binding site
Cbf1bs_top	T CACGTGA TCACGTGA T CACGTGA	Oligo used for cloning Cbf1 binding site
Cbf1bs_bottom	TCACGTG ATCACGTGA TCACGTG A	Oligo used for cloning Cbf1 binding site
Gcr1bs_top	TGGAAGCCTTGGAAGCCTTGGAAGCCT	Oligo used for cloning Gcr1 binding site
Gcr1bs_bottom	AGGCTTCCAAGGCTTCCAAGGCTTCCA	Oligo used for cloning Gcr1 binding site
Cat8bs_top	CCTTTAAGCCGACCTTTAAGCCGACCTT TAAGCCGA	Oligo used for cloning Cat8 binding site
Cat8bs_bottom	TCGGCTTAAAGGTCGGCTTAAAGGTCG GCTTAAAGG	Oligo used for cloning Cat8 binding site
